# Neutrophils Return to Bloodstream Through the Brain Blood Vessel After Crosstalk With Microglia During LPS-Induced Neuroinflammation

**DOI:** 10.3389/fcell.2020.613733

**Published:** 2020-12-08

**Authors:** Yu Rim Kim, Young Min Kim, Jaeho Lee, Joohyun Park, Jong Eun Lee, Young-Min Hyun

**Affiliations:** ^1^Department of Anatomy, Yonsei University College of Medicine, Seoul, South Korea; ^2^BK21 PLUS Project for Medical Science, Yonsei University College of Medicine, Seoul, South Korea; ^3^Department of Medicine, Yonsei University College of Medicine, Seoul, South Korea

**Keywords:** neuroinflammation, neutrophil, microglia, reverse transendothelial migration, two-photon intravital imaging

## Abstract

The circulatory neutrophil and brain tissue-resident microglia are two important immune cells involved in neuroinflammation. Since neutrophils that infiltrate through the brain vascular vessel may affect the immune function of microglia in the brain, close investigation of the interaction between these cells is important in understanding neuroinflammatory phenomena and immunological aftermaths that follow. This study aimed to observe how morphology and function of both neutrophils and microglia are converted in the inflamed brain. To directly investigate cellular responses of neutrophils and microglia, LysM^GFP/+^ and CX_3_CR1^GFP/+^ mice were used for the observation of neutrophils and microglia, respectively. In addition, low-dose lipopolysaccharide (LPS) was utilized to induce acute inflammation in the central nervous system (CNS) of mice. Real-time observation on mice brain undergoing neuroinflammation via two-photon intravital microscopy revealed various changes in neutrophils and microglia; namely, neutrophil infiltration and movement within the brain tissue increased, while microglia displayed morphological changes suggesting an activated state. Furthermore, neutrophils seemed to not only actively interact with microglial processes but also exhibit reverse transendothelial migration (rTEM) back to the bloodstream. Thus, it may be postulated that, through crosstalk with neutrophils, macrophages are primed to initiate a neuroinflammatory immune response; also, during pathogenic events in the brain, neutrophils that engage in rTEM may deliver proinflammatory signals to peripheral organs outside the brain. Taken together, these results both show that neuroinflammation results in significant alterations in neutrophils and microglia and lay the pavement for further studies on the molecular mechanisms behind such changes.

## Introduction

Neuroinflammation is generally defined as the response of brain cells to infection and other sources of cell death, involving infiltration of circulating immune cells to the brain. Such infiltration of immune cells occur due to microglial and glial cell activation and blood-brain barrier (BBB) dysfunction during the pathogenesis of various illnesses, such as Alzheimer’s disease, Parkinson’s disease, and Amyotrophic lateral sclerosis (Shastri et al., 2013; Mammana et al., 2018).

Neutrophils are commonly known as the earliest responders to acute inflammation, aiding the initiation and continuation of immune reactions throughout the human body (Nathan, 2006). Neutrophils are highly versatile cells with various immune functions, such as inflammation mediation, microbial capture via granular proteins, and repair of sterile wounds (Kruger et al., 2015). In particular, during neuroinflammation, neutrophils participate in the general immune response by signaling to diverse cell types, including endothelial cells, mesenchymal stem cells, lymphocytes, and microglia (Ahn et al., 2020). Recent reports have emphasized the variety of roles neutrophils can play in neuroinflammation, where brain resident cells participate in a coordinated fashion (Liu et al., 2018).

Microglia, the resident macrophages of the central nervous system (CNS), are distinguished from other glial cells, such as astrocytes and oligodendrocytes by their gene expression, morphology, and function (Ransohoff and Perry, 2009; Kettenmann et al., 2011; Zhao et al., 2019). In contrast to other glial cells, microglia function as the primary reacting cells for regulating neuroinflammatory response by phagocytizing and removing myelin inhibitors, debris and dead cells in the CNS (Li et al., 2005; Zhao et al., 2019). Microglia also take part in innate and adaptive immunity by regulating immune tolerance (Saijo and Glass, 2011). Microglia are functionally and morphologically divided into three forms: the ramified, activated and ameboid morphologies. Ramified microglia, with a small cell body and long branches, have no functional capability of phagocytosis and antigen presentation but maintain an immunologically stable environment. When ramified microglia are stimulated by neurodegeneration, endotoxin, interferon, or endothelial activation, activation pathways cause them to transform into activated microglia. Activated microglia exhibit thicker and more retracted branches and possess the ability exhibit antigen presentation and phagocytosis. Additionally, activated microglia, when changed to the ameboid shape, display free movement during phagocytosis but do not engage in antigen presentation and inflammation (Cai et al., 2014). In addition, excessive or long-term activation of microglia induces neuronal death and an increase in pro-inflammatory cytokines.

In this study, we aimed to observe the effects of lipopolysaccharide (LPS)-induced neuroinflammation on neutrophils and microglia within brain tissue of live mice. To this end, we attempted to obtain visual evidence of the effects of neuroinflammation on neutrophils and microglia using two-photon intravital imaging, which may then serve as a basis for further research on the molecular and mechanistic bases of such modifications.

## Materials and Methods

### Mice

LysM^GFP/+^ (Faust et al., 2000) and CX_3_CR1^GFP/+^ (Jung et al., 2000) mice, in which the lysozyme and the CX_3_CR1 gene are replaced by green fluorescent protein (GFP), respectively, were obtained and used for the visualization of neutrophil and microglia. All mice were kept in a specific pathogen-free (SPF) room, a light cycle from 7:00 AM to 7:00 PM at 23 ± 2°C, and 55 ± 10% humidity. All procedures were conducted in accordance with the guidelines of the Institutional Animal Care and Use Committee of Yonsei University College of Medicine, South Korea (IACUC, 2019-0097).

### Identification of Mouse Genotype

Genotyping for each strain (LysM^GFP/+^ and CX_3_CR1^GFP/+^ mice) was performed using a Genomic DNA Prep Kit (BioFACT, South Korea). A toe of 7–10 day-old mice was severed, and then DNA extraction from the acquired toe was conducted using the Genomic DNA Prep Kit. Template DNA (50 ng/μl), primers and 2×Taq PCR master mix2 10 μl (BioFACT, South Korea) were mixed in a PCR tube, in which distilled water was added up to 20 μl reaction volume.

### Cranial Window Surgery

The cranial window was implanted on the calvaria for intravital brain imaging as previously described (Baik et al., 2014). Mice were deeply anesthetized using intraperitoneal injection of zoletil (Virbac, France) at a dose of 30 mg/kg and rompun (Bayer, Germany) at a dose of 10 mg/kg. Body temperature in each mouse was maintained at 37 ± 0.5°C using heating pads during cranial window surgery (Supplementary Figure 1). Mice were fixed in a stereotaxic instrument (Live Cell Instrument, South Korea) during all procedures. A cranial window of 5 mm in diameter was made in the right hemisphere. The head skin and the periosteum on the calvaria were removed from between the eyes to the caudal region of the ears. Between the lambda and bregma regions on the right hemisphere, a circular opening was carved with a micro drill, frequently washed with cool phosphate-buffered saline (PBS), and sealed with a round coverslip (diameter = 5 mm) using tissue glue on the skull using the optical microscope. A metal frame was glued and fixed using dental cement (B.J.M laboratory, Israel) on the borders of the cranial window and skull area for filling imaging area with distilled water. The metal frame was assembled with a stereotactic head fixation device attached to the heating plate.

### Two-Photon Intravital Microscopy

Mice were anesthetized using intraperitoneal injection of Zoletil at a dose of 30 mg/kg and rompun at a dose of 10 mg/kg during imaging. The imaging stage was composed of a XY micro stage and a stereotactic head fixation device connected to a DC temperature controller (Supplementary Figure 1A). Both two-photon microscopies with W Plan-Apochromat 20×/1.0 water immersion lens from Carl-Zeiss, Germany (LSM7MP) and from IVIM Technology, South Korea (IVM-M) were used for imaging data generation. LysM^GFP/+^ mice were intravenously injected with 70-kDa Texas red dextran (2.5 mg/kg, Sigma-Aldrich, Germany) for visualizing blood vessels. CX_3_CR1^GFP/+^ mice were intravenously injected with CF405M-conjugated Wheat germ agglutinin (WGA) (2.5 mg/kg, Biotium, CA, United States) for visualizing blood vessels and PE-conjugated anti-Ly6G antibody (0.1 mg/kg, BioLegend, CA, United States) for observing neutrophils. For two-photon excitation, each mouse brain was excited with light of 800 nm and 880 nm wavelength for imaging of green, red, and blue. All images were acquired at a resolution of 512 × 512 pixels using steps of size 1 μm to a depth of 40–50 μm for 1 min (Park et al., 2018; Lee et al., 2019).

### LPS-Induced Neuroinflammatory Mouse Model

Previous studies established that LPS-induced inflammation in the periphery can prompt immune responses in the central nervous system (Ebersoldt et al., 2007; Zhao et al., 2019). To investigate the migratory patterns of neutrophil and microglia during neuroinflammation, mice were treated with daily intraperitoneal injections of lipopolysaccharide (1.0 mg/kg, Sigma-Aldrich, Germany) for 2 consecutive days. Control mice were intraperitoneally injected with daily PBS injections for 2 consecutive days. Intravital imaging was performed at 6 h after LPS injections for 2 consecutive days.

### Imaging Data Analysis

Volocity (PerkinElmer, MA, United States), Imaris (Bitplane, Switzerland), and Fiji/Image J (NIH, United States) were used for 3D and 4D imaging data analysis.

### Chemokine Microarray

Following LPS stimulation, brains of mice were lysed by adding protease inhibitor cocktail (Roche, Germany) containing PRO-PREP (Intron biotechnology, South Korea) and 1% Triton X-100. Cytokine and chemokine levels were detected using Proteome Profiler Mouse Cytokine Array Panel A (R&D systems, MN, United States) according to the manufacturer’s instruction. The Array kit detected C5/C5a, G-CSF, M-CSF, GM-CSF, sICAM-1, IFN-γ, IL-1α, IL-1β, IL-1ra, IL-2, IL-3, IL-4, IL-5, IL-6, IL-7, IL-10, IL-13, IL-12p70, IL-16, IL-17, IL-23, IL-27, CXCL1, CXCL2, CXCL9, CXCL10, CXCL11, CXCL12, CXCL13, CCL1, CCL2, CCL3, CCL4, CCL5, CCL11, CCL12, CCL17, TIMP-1, TNF-α, and TREM-1. The blots were analyzed using the quick spots tool in HLImage++ (Western Vision Software, UT, United States).

### Statistical Analysis

All experiments were repeated at least three times. Statistical analyses were expressed as mean ± standard error of the mean (S.E.M). Statistical analysis was performed using Prism (GraphPad software, CA, United States). For comparison of two groups, unpaired two-sided Student *t*-tests were applied. *p*-values less than 0.05 were considered statistically significant.

## Results

### LPS-Induced Systemic Inflammation Triggers Intravascular Adhesion and Infiltration of Neutrophils Through Brain Blood Vessels

**FIGURE 1:**
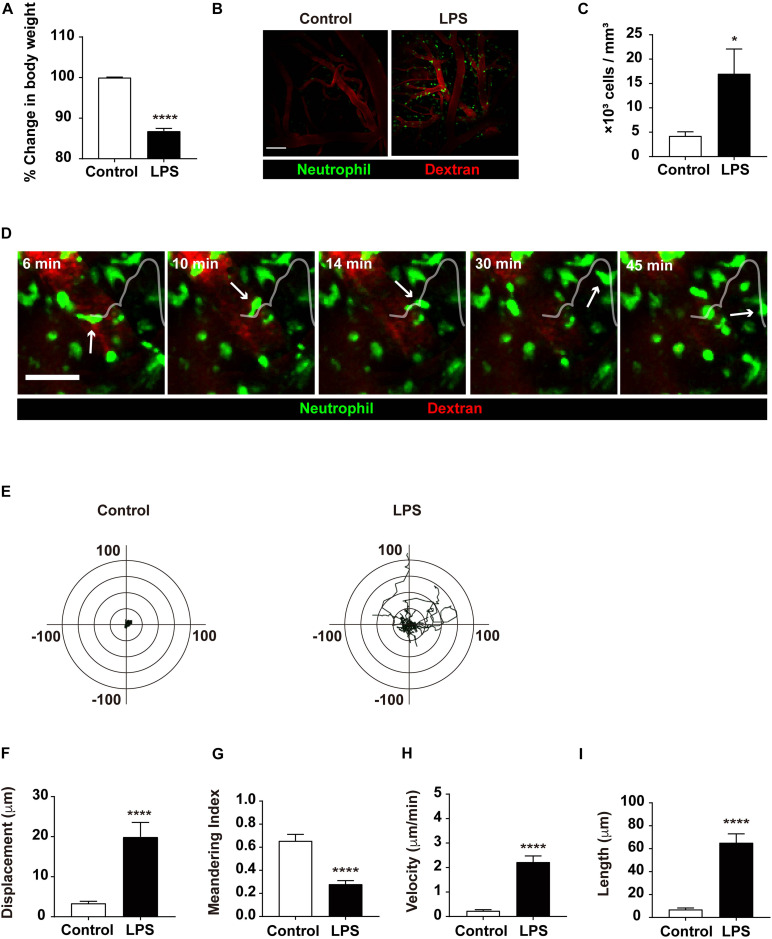
Quantitative analysis of neutrophil infiltration from the blood vessel to brain parenchyma. **(A)** Representative graph of weight loss in the LPS-treated group compared to the control group. The % change in body weight was calculated as (body weight before LPS treatment/body weight after LPS treatment) × 100. Data indicate mean ± SEM using Student’s *t*-test (****p < 0.0001, *n* = 7 mice per group). **(B)** LysM^GFP/+^ mice were used to visualize neutrophils (green) via TPIM. Texas red dextran was i.v. injected to stain blood vessels (red). Representative images are shown from the brain of mice injected with PBS (control) or LPS, respectively. Scale bar, 100 μm (see Supplementary Video 1). **(C)** Measurement of infiltrated neutrophils in the control and LPS groups. Data indicate mean ± SEM using Student’s *t*-test (*p < 0.05, *n* = 3 mice per group of three independent experiments). **(D)** A series of representative time-lapse images show neutrophil infiltration into inflamed brain parenchyma of LysM^GFP/+^ mice. Scale bar, 50 μm (see Supplementary Video 2). **(E)** Overlay of the representative migration tracks of neutrophils in brain parenchyma for 30 min. *x*-, *y*-axis (length), -100 to 100 μm. *n* = 30 cells per group, PBS or LPS-treated mice for three independent experiments. Migration was quantitatively assessed with tracking analyses: **(F)** displacement (μm), **(G)** meandering index (displacement/length), **(H)** velocity (μm/min), and **(I)** length (μm) in two different conditions for 30 min. Data indicate mean ± SEM using Student’s *t*-test (****p < 0.0001, *n* = 30 cells per group of three independent experiments).

To investigate the effect of inflammatory status via LPS injection, the weight of mice injected with LPS was compared to that of the control group, as weight loss is a hallmark of systemic inflammation. The LPS group showed an 11.75% loss in body weight compared to the control group, confirming that inflammation had indeed been induced in the LPS-injected mice (Figure 1A). Previous studies have demonstrated that neutrophils are recruited in the brain during LPS-induced systemic inflammation to fulfill their roles in the immune response (Zhou et al., 2009; He et al., 2016). Our results confirmed this result, as neutrophil extravasation to the brain parenchyma was observed more frequently in response to LPS injection. In addition, an increased number of neutrophils were observed, which resulted from intravascular adhesion and infiltration (Figures 1B,C and Supplementary Video 1). Consistent with such findings, transendothelial migration (TEM) of neutrophils was also facilitated in the LPS group, where neutrophils actively emerged out to the brain parenchyma (Figure 1D and Supplementary Video 2). Altogether, these data demonstrate that LPS injection and the subsequent inflammatory consequences that follow yield a considerable increase in neutrophil influx to the brain parenchyma.

### Neutrophils Exhibit Active Motility in the Brain Parenchyma During Neuroinflammation

Along with an increment in cell number, neutrophils displayed an increase in motility, as exhibited in various motion-related criteria. The motility of neutrophils was determined and assessed using various factors, including track length, track velocity, displacement, and meandering index. The track length and track velocity of migrating neutrophils in the brain parenchyma were significantly higher in the LPS group compared to the control group, indicating more active locomotion in response to LPS injection. Furthermore, these results revealed that infiltrated neutrophils showed constant migration within a 20 μm radius for 30 min, suggesting significant motility of neutrophils during neuroinflammatory response; in addition, a lower meandering index compared to the control group may signify more directionality in neutrophil movement in LPS-injected mice (Figures 1E–I). Thus, these results indicate that during neuroinflammation, the motility of neutrophils in the brain parenchyma is notably increased, suggesting a change in the molecular and biochemical profile of the neutrophils.

### Inflammation in the Brain Triggers Numerical Reduction and Morphological Shortening of Microglia

**FIGURE 2:**
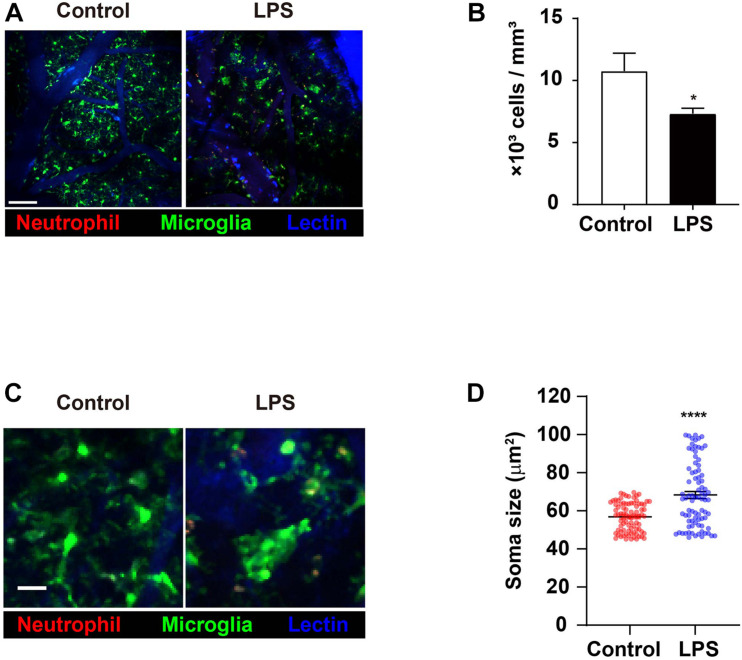
Numerical reduction and morphological shortening of microglia in LPS-treated mice. CX_3_CR1^GFP/+^ mice were used to visualize microglia (green) in brain parenchyma using TPIM. CF405M-conjugated lectin was i.v. injected to stain blood vessels (blue). PE-conjugated anti-Ly6G antibody was i.v. injected to label neutrophils (red). **(A)** Representative images from brains of mice injected with PBS (control) and LPS, respectively (see Supplementary Video 3). Scale bar, 100 μm. **(B)** Measurement of microglia numbers. Data indicate mean ± SEM using Student’s *t*-test (*p < 0.05, *n* = 3 mice per group of three independent experiments). **(C)** Representative image showing difference in microglial morphologies between control and LPS groups. Scale bar, 30 μm (see Supplementary Video 4). **(D)** Quantification of microglia soma size. Data indicate mean ± SEM using Student’s *t*-test (****p < 0.0001, more than 70 cells were analyzed per group from three independent experiments).

As the predominant innate immune cell population that is resident to the brain, microglia play a role in the process of neuroinflammation. Therefore, it was important that the effects of LPS injection and the ensuing inflammatory aftermaths on microglia were investigated. Specifically, it had previously been suggested that neutrophils may interact with microglia; as our aforementioned results indicate various changes in neutrophils during neuroinflammation, it is crucial to unravel any microglial changes during similar situations in order to pinpoint any biomolecular interactions between the two innate immune cell populations (Sevenich, 2018). Imaging data in the brain showed that the number of microglia in LPS-injected mice had decreased compared to the control group, a phenomenon that may have been caused by a variety of apoptosis-inducing agents (Figures 2A,B and Supplementary Video 3; Steff et al., 2001; Fortin et al., 2005). Also, while microglia in the control group predominantly displayed the morphology of ramified microglia, those in the LPS group showed the morphology of activated microglia, with short and thick processes and enlarged cell bodies (Figure 2C and Supplementary Video 4). To focus on a more detailed analysis, such as the microglia cell soma, we quantified the change in microglia soma size between control and LPS group. Compared to control mice, the average of soma size increased (56.85 vs. 68.32 μm^2^) (Figure 2D). The result demonstrated the LPS exposure leads to activated microglia that features bigger soma size than ramified microglia called “resting” cells (Kozlowski and Weimer, 2012; Davis et al., 2017). Taken together, these results demonstrate that LPS-induced neuroinflammation may activate microglia, leading to morphological alterations that suggest an activated state (Liu and Hong, 2003).

### Neutrophil-Microglia Crosstalk in Inflamed Brain Parenchyma Leads to Engulfment of Neutrophils by Microglia

**FIGURE 3:**
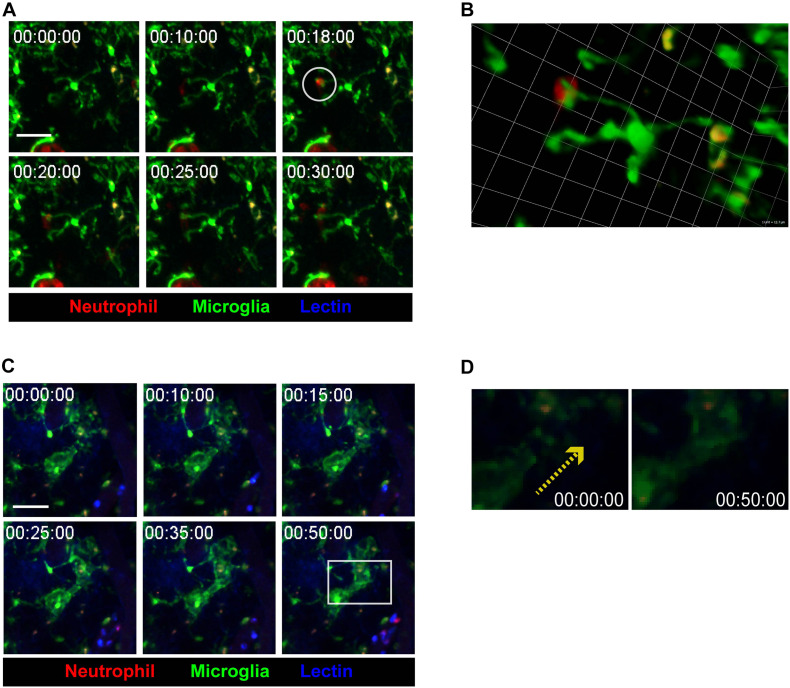
Crosstalk between microglia and neutrophils in inflamed brain parenchyma. CX_3_CR1^GFP/+^ mice were used to visualize microglia (green) in brain parenchyma using TPIM. CF405M-conjugated lectin was i.v. injected to stain blood vessels (blue). PE-conjugated anti-Ly6G antibody was i.v. injected to label neutrophils (red). **(A)** A series of representative time-lapse images showing contact between microglia and neutrophils (white circle). Scale bar, 40 μm (see Supplementary Video 5). **(B)** Magnification of a region of interest, indicated by a white circle in **(A)**, in 3D. Scale bar per 1 unit. Scale bar, 12.7 μm. **(C)** A series of representative time-lapse images showing elongation of microglial processes toward site neutrophil-microglial contact (white square). Scale bar, 40 μm (see Supplementary Video 6). **(D)** Direction of microglial process elongation from 0 to 50 min (yellow-dotted arrow).

During the neuroinflammatory response, neutrophils have been thought to actively interact with cells in the vicinity, such as astrocytes, microglia, and adaptive immune cells (He et al., 2016; Girbl et al., 2018). Imaging data from the present study supports this idea, as contact between infiltrated neutrophils and brain tissue-resident microglia was observed (Figures 3A,B and Supplementary Video 5). After neutrophils made contact with microglial processes, microglia seemed to engulf the neutrophils, indicating the possibility of molecular crosstalk between the two cell groups. On the other hand, during neutrophil-microglial contact, nearby microglial processes were observed to stretch toward the point of contact (Figures 3C,D and Supplementary Video 6). Such phenomena further reinforces the idea that contact between neutrophil and microglia accompanies intercellular crosstalk, affecting not only the cells making contact but also the surrounding environment. Overall, while the underlying mechanisms are yet to be revealed, these results strongly suggest that inflammatory states not only prompt crosstalk and contact between neutrophils and microglia, but also attract other microglia toward sites of neutrophil-microglia contact.

### Reverse Transendothelial Migration of Neutrophils Is Observed in Inflamed Brain Parenchyma

**FIGURE 4:**
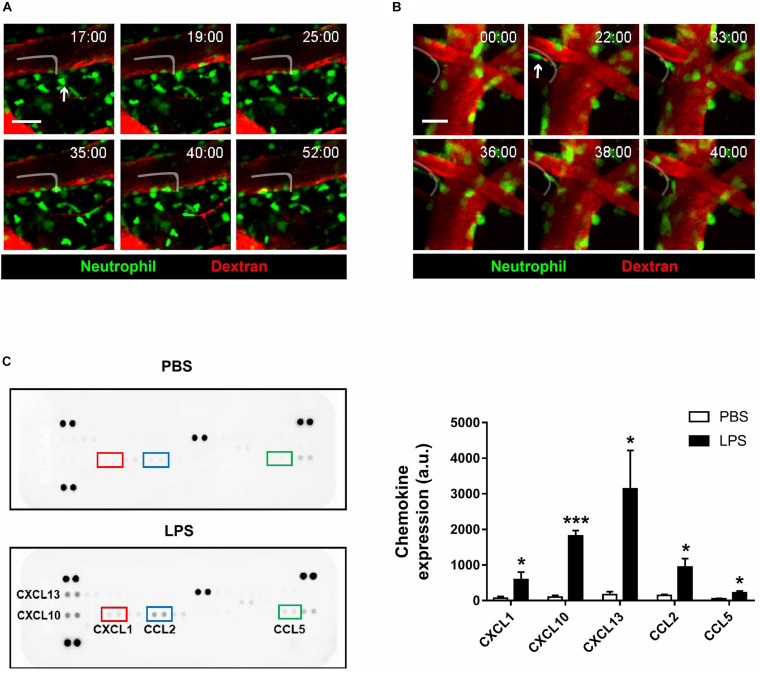
Reverse-transendothelial migration of neutrophils in inflamed brain parenchyma. LysM^GFP/+^ mice were used to visualize neutrophils (green) during neuroinflammation. Texas red dextran was i.v. injected to stain blood vessels (red). **(A)** A series of representative time-lapse images showing neutrophil crawling along the perivascular region in inflamed brain parenchyma. Tracking of neutrophil (white line). Scale bar, 50 μm (see Supplementary Video 7). **(B)** A series of representative time-lapse images showing reverse-transendothelial migration of neutrophils from brain parenchyma into blood vessels. Tracking of neutrophil (white line). Scale bar, 20 μm. **(C)** Brain chemokine array results demonstrating altered expression levels of chemokines following LPS injection. Data indicate mean ± SEM using Student’s *t*-test (*p < 0.005, ***p < 0.001). Three independent experiments were performed.

An interesting event regarding neutrophils that have been reported in previous literature is reverse transendothelial migration (rTEM), where neutrophils that had extravasated out of the blood vessel re-enters the bloodstream (Colom et al., 2015; Wu et al., 2016; Burn and Alvarez, 2017). While rTEM had been observed in various parts of the body, imaging results from the present study are the first to suggest that the process also takes place in brain blood vessels (Figures 4A,B and Supplementary Videos 7, 8). Neutrophils engaging in rTEM first approach the blood vessel, and after a period of movement along the perivascular region, re-enters and eventually gets swept away by the bloodstream. Albeit a rare phenomenon, rTEM of neutrophils that had been exposed to inflammatory environments may lead to significant repercussions, as it is possible that the molecular profile of re-entering neutrophils is altered; nevertheless, the present results suggest that rTEM is a readily observable phenomenon during neuroinflammation.

To investigate the molecular aftereffects of LPS injection that may have stimulated neutrophil rTEM, brain chemokine arrays were conducted to detect changes in chemokine expression levels following LPS injection (Figure 4C). As a result, levels of the chemokines CXCL1, CXCL10, CXCL13, CCL2, and CCL5 were found to be increased, a consequence which may affect the function and motility of microglia and neutrophils. As such, it may be postulated that increased levels of the aforementioned chemokines had stimulated neutrophil migration and rTEM as presented above.

## Discussion

The CNS had been known to be an “immune privileged site,” most likely due to the presence of the highly impenetrable BBB; yet recent studies have demonstrated flexibility in the BBB in response to inflammation-related needs and stimuli (Kanashiro et al., 2020). Indeed, in neuroinflammatory situations, neutrophils and CNS-resident microglia are suggested to participate in the inflammatory response, as supported by the imaging results of this study. With such visual evidence at hand, investigation of the molecular bases behind the observed phenomena is of significant importance, especially in clinical settings. In previous literature, the molecules responsible for the recruitment of neutrophils to neuroinflammatory sites have been given substantial attention. For instance, in the case of autoimmune diseases, such as multiple sclerosis or experimental autoimmune encephalomyelitis, neutrophils are known to be attracted by the release of CXCL1, CXCL2, and CXCL5, which are in turn stimulated by different molecules, such as IL-17 or IFN-γ (Christy et al., 2013; Simmons et al., 2014). The behavior of neutrophils in neurodegenerative diseases including Alzheimer’s has also been a target of scrutiny, as neutrophil infiltration is known to contribute to the exacerbation of such diseases. In particular, it has been suggested that neutrophils accumulate in regions rich in amyloid-β deposits via the integrin LFA-1; in addition, CXCL12 and CCL2 levels in the CNS were shown to be principal factors resulting in neutrophil infiltration (Zenaro et al., 2015; Echeverria et al., 2016; Jiang et al., 2016). In line with results from previous literature, expression levels of certain chemokines were increased after LPS injection in this study; in specific, among the aforementioned chemokines, CXCL1 and CCL2 demonstrated a surge in expression, along with CXCL13, CXCL10, and CCL5. CXCL1 and CCL2 are well-known for their roles in stimulating neutrophil migration, while CXCL10, CCL2, and CCL5 have been reported to be released by neutrophils in inflammatory situations, mostly in order to recruit other innate or adaptive immune cells (Kobayashi, 2008; Sawant et al., 2016; Capucetti et al., 2020). Therefore, chemokines that are chemotactic for neutrophils, such as CXCL1 or CCL2 may be the principal factor driving the various migratory phenomena observed in this paper: neutrophil extravasation and rTEM. In addition, it may be postulated that LPS-induced neuroinflammation had caused phenotypical modifications in neutrophils, prompting them to release chemokines that yield further steps down the inflammatory cascade, including further recruitment of neutrophils or chemoattraction of adaptive immune cells. On the other hand, CXCL13 plays a role in adaptive immune cell organization, being especially chemotactic for B cells; yet, it is not known to be expressed in neutrophils (Havenar-Daughton et al., 2016).

One of the most interesting phenomena that deserve attention in the present study is rTEM, the reverse migration of neutrophils from the brain parenchyma back to the bloodstream. Although its exact purpose and mechanism are yet to be clearly defined, rTEM has been observed in numerous previous studies, and accumulation of relevant data is continuing to provide new insight into the topic (Burn and Alvarez, 2017). For instance, several studies have attempted to pinpoint the molecular signals that seem to induce or influence rTEM; in one study, cold-inducible RNA-binding protein (CIRP) has been suggested to stimulate neutrophil rTEM in septic conditions via an increase in neutrophil elastase and a decrease in junctional adhesion molecule-C (JAM-C) (Jin et al., 2019). In addition, the chemoattractant leukotriene B_4_ (LTB4) has been proposed to be a potential factor which causes proteolytic cleavage of JAM-C via neutrophil elastase, further reinforcing the possible roles of neutrophil elastase and JAM-C in rTEM (Colom et al., 2015). Along with JAM-C, netrin-1, a protein highly expressed in endothelial cells and is known to disrupt leukocyte transendothelial migration, has also been proposed as a factor that may hinder rTEM (Ly et al., 2005; Podjaski et al., 2015). In particular, it has been shown that netrin-1 activation is dependent on hypoxia inducible factor 1 alpha (Hif-1α), which in turn seems to promote continuation of inflammation and prevent neutrophil clearance from perivascular regions (Rosenberger et al., 2009; Elks et al., 2011). Furthermore, Tanshinone IIA, a compound originated from an Asian medicinal herb, has been shown to promote inflammation resolution via the induction of neutrophil apoptosis and rTEM (Robertson et al., 2014). Yet, as Tanshinones have also been suggested to play an inhibitory role on NFkB, AP-1, and STAT1 activation and thus possess anti-inflammatory functions, additional research on the matter is necessary to confirm the role of Tanshinones in the context of rTEM and neutrophil clearance (Xu et al., 2008; Tang et al., 2011).

Yet while the molecular mechanisms underlying rTEM is crucial, another important issue is perhaps the consequences of neutrophil rTEM, especially the effects of the neutrophils returning to the bloodstream on other organs. Post-rTEM neutrophils were suggested to play diverse roles, including inhibition of T cell proliferation, neutrophil clearance from tissues, enhancement of reactive oxygen species (ROS) and neutrophil extracellular trap (NET) formation, and exacerbation of systemic inflammation (Buckley et al., 2006; Mathias et al., 2006; Woodfin et al., 2011; Yoo and Huttenlocher, 2011; Brinkmann and Zychlinsky, 2012; Pillay et al., 2012; Cheng and Palaniyar, 2013). Notably, the molecular profile of neutrophils that had underwent rTEM exhibited high levels of CD11b, CD54, and ICAM-1, while showing low levels of CD62L, CXCR1, and CXCR2 markers (Walcheck et al., 1996; Buckley et al., 2006; Woodfin et al., 2011). The distinguishable molecular expression pattern in neutrophils that had returned to the bloodstream presents various inquiries, including what effects such patterns may have on the function of neutrophils after rTEM. Another important question that arises would be how those neutrophils acquired such a state; one likely hypothesis is that the neutrophils experienced molecular modification via active interaction with other cells. For example, it had been postulated that the high expression of ICAM-1 on the surface of post-rTEM neutrophils may have resulted from a mechanism that resembles trogocytosis, by which neutrophils acquired ICAM-1 high membranes from endothelial cells (Joly and Hudrisier, 2003; Burn and Alvarez, 2017).

In this context, it is natural to speculate whether neutrophils that had been chemically stimulated or modified by interactions with microglia engage in rTEM, thereby spreading inflammatory signals from the brain to other peripheral organs of the body. Although the accumulation of neutrophils in inflammatory conditions is a well-established concept, the interaction between neutrophils and microglia during neuroinflammation has yet to be extensively studied. Our study presents compelling evidence of neutrophil-microglial contact, which also led to further mobilization of nearby microglia toward the site of contact. Previously, the same phenomenon had been observed via two-photon imaging in a stroke model, where neutrophils infiltrated the brain parenchyma following cortical ischemia and microglia engulfed the neutrophils, in line with the results of the present study (Neumann et al., 2018). The fact that identical observations were made in neuroinflammatory situations via different inducers (systemic inflammation via LPS injection vs. ischemic stroke) demonstrates that neutrophil-microglia contact might be a general phenomenon in neuroinflammation, raising questions regarding the molecular mechanisms or cell signaling underlying such interactions. Despite the fact that studies on such topics are scarce, previous research has suggested that molecules, such as RGD peptides of GlcNAC may hinder microglia-neutrophil interactions *in vitro*, possibly laying the pavement for further mechanistic studies (Neumann et al., 2008). Furthermore, previous research on neutrophil rTEM indicate that the majority of neutrophils that engage in rTEM had prior interaction with macrophages, strongly suggesting a role of macrophages promoting neutrophil rTEM not only in the brain but also in other organs in general (Burn and Alvarez, 2017). In particular, the redox-SRC family kinase (SFK) signaling pathway was shown to be relevant to the rTEM-inducing capabilities of macrophages, with p22phox and Yes-related kinases being key players (Tauzin et al., 2014). While contact with macrophages was not necessary for neutrophil rTEM, the number of neutrophils that undergo rTEM significantly diminished in a setting that lacked macrophages; such previous data, combined with the results of the present study, presents the need to delve further into the impact of neutrophil-macrophage interactions on neutrophil rTEM. Meanwhile, combining the present results with observations from previous literature, we may even speculate that the neutrophils that had interacted with microglia in the brain parenchyma had underwent molecular modifications that predispose them to engage in rTEM; in this sense, neutrophil-microglia crosstalk may be crucial in the resolution of inflammation in the brain.

One key discrepancy between previous studies on neutrophil rTEM and the present study is the rate in which rTEM was observed; although it had been proposed that up to 80% of the neutrophils that accumulated in inflammatory sites may engage in rTEM, the phenomenon was not seen as frequently in the brain (Mathias et al., 2006; Yoo and Huttenlocher, 2011). This may be possibly due to the complexity and relative impenetrability of the BBB, which often results in various complications regarding leukocyte migration (Ransohoff et al., 2003). In this context, further research is necessary to investigate why rTEM is observed in a much lower frequency in the brain. At the same time, the occurrence of rTEM, however rare as it may be, also presents a need to decipher the brain-specific mechanisms behind rTEM through the BBB. The aforementioned netrin-1 may be a reasonable starting point, as the molecule is known to be involved in BBB function (Podjaski et al., 2015).

**FIGURE 5:**
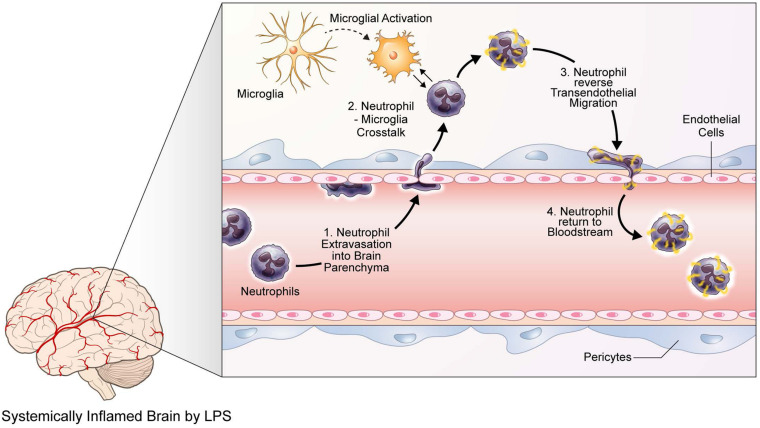
Neutrophils and microglia exhibit various behavioral and phenotypical responses to LPS-induced neuroinflammation. A graphic illustration describing the responses of neutrophils and microglia to LPS-induced neuroinflammation, as identified via two-photon microscopy. Upon LPS injection and the following neuroinflammatory situations, neutrophils first respond by extravasating into the brain parenchyma. Meanwhile, microglia are activated and thus undergo morphological changes, such as enlargement of cell bodies and shortening of processes. Such activated microglia interact with infiltrated neutrophils, possibly causing molecular modifications on the neutrophils; thereafter, neutrophils engage in reverse transendothelial migration, returning to the bloodstream.

In the present study, we have demonstrated that neuroinflammation induced by LPS injection yields increased recruitment and mobility of neutrophils in the brain parenchyma, while activating microglia, as observed from their morphological changes. In addition, neutrophils that had infiltrated brain tissue were seen to engage in neutrophil-microglia interaction, where microglia engulfed the neutrophils in contact. Such a phenomenon also attracted nearby microglia, inducing dendritic movement toward the site of contact. Furthermore, infiltrated neutrophils also exhibited rTEM, returning to the bloodstream after entering the brain parenchyma (Figure 5). Despite such compelling evidence, this paper possesses several limitations. First and foremost, it would benefit greatly from further research that is able to observe rTEM at a higher frequency, as one of the biggest gaps between data in this paper and data on rTEM studies was that between the rate of rTEM observation. Another limitation is that in this study, we were not able to quantify meaningful ratios and proportions of cells exhibiting desired behavior. For instance, the ratio of neutrophils that engaged in extravasation to those that circulated within the blood vessel, the ratio of neutrophils that infiltrated to the brain parenchyma to those that interacted with microglia, and the ratio of neutrophils that interacted with microglia to those that exhibited rTEM are all of much importance but were unattainable due to technological issues. Also, as most of the results of this paper are of an observational nature, future studies utilizing *in vivo* or *in vitro* methods may assist in underpinning the biomolecular mechanisms behind the observations of this paper, specifically in relation to neutrophil-microglial interactions. Finally, while the current study utilized LPS injection to induce neuroinflammation, the variety of neuroinflammatory models existing today will be of tremendous usefulness in verifying the present results, in perhaps a more clinical setting. For example, in our previous data, we had demonstrated neutrophil infiltration into the brain parenchyma and accumulation around amyloid beta plaques in 5XFAD mice, an AD mouse model, using two-photon microscopy; Zenaro et al., later confirmed similar results in 3×Tg-AD mice, another murine model of AD. As such, further studies that corroborate the results of the present study using disease-specific models of neuroinflammation may help investigating the clinical implications of neutrophil infiltration, neutrophil-microglia interaction, and neutrophil rTEM in neuroinflammation. Taking such future research suggestions into consideration, this paper presents strong visual data on the behavior of neutrophils in response to neuroinflammatory situations and may establish the groundwork for future research on the molecular mechanisms underlying innate immune cell responses to neuroinflammation and systemic inflammation alike.

## Data Availability Statement

The original contributions presented in the study are included in the article/Supplementary Material, further inquiries can be directed to the corresponding author/s.

## Ethics Statement

The animal study was reviewed and approved by the Institutional Animal Care and Use Committee of Yonsei University College of Medicine, South Korea.

## Author Contributions

YRK performed the experiments, wrote the manuscript, and analyzed the data. YMK wrote the manuscript and analyzed the data. JL and JP performed the experiments. JEL helped design the study. Y-MH directed the study design and wrote the manuscript. All authors contributed to the article and approved the submitted version.

## Conflict of Interest

The authors declare that the research was conducted in the absence of any commercial or financial relationships that could be construed as a potential conflict of interest.

## Supplementary Material

Supplementary Figure 1 Cranial window surgery setup for brain intravital imaging. **(A)** Brain chambers with heating plates for maintaining body temperature at 37 ± 0.5°C. **a.** Metal and rubber heating plates, **b.** DC temperature controller, **c.** Stereotactic instrument for cranial window surgery, **d.** Head fixation device for imaging. **(B)** Subsidiary tools for surgery **a.** cotton swab, **b.** air blower, **c.** tissue glue, **d.** cover glass (diameter = 5 μm), **e.** metal frame, **f.** pen, **g.** dental cement, **h.** resin tip, **i.** forceps, **j.** a pair of scissors, **k.** forceps, **l.** micro forceps, **m.** micro drill. **(C)** Surgical procedure of cranial window surgery for intravital brain imaging. **i.** Fix the mouse in a stereotactic heating plate, **ii.** Remove head skin and the periosteum, **iii.** Coordinate predestinate location carved on right hemisphere, **iv.** Moisturize a circular opening with a drop of isotonic saline solution and seal a circular opening with a 5 μm coverslip, **v.** Glue and fix the metal frame on the borders of the cranial window and skull area using dental cement, **vi.** Place the mouse on a head fixation device for intravital imaging.

Supplementary Video 1 TPIM of brain parenchyma in LysM^GFP/+^ mice injected with PBS (control) and LPS, respectively. Green, neutrophil (GFP); Red, blood vessels (Texas Red dextran). Scale bar, 100 μm.

Supplementary Video 2 TPIM of neutrophil infiltration during neuroinflammation. Green, neutrophil (GFP); Red, blood vessels (Texas Red dextran); White, track line of neutrophil. Scale bar, 50 μm.

Supplementary Video 3 TPIM of brain parenchyma in CX_3_CR1^GFP/+^ mice injected with PBS (control) and LPS, respectively. Green, microglia (GFP); Red, neutrophils (Ab Ly6G); Blue, blood vessels (Lectin). Scale bar, 100 μm.

Supplementary Video 4 TPIM of morphological changes in microglia in brain parenchyma. Green, microglia (GFP); Red, neutrophils (Ab Ly6G); Blue, blood vessels (Lectin). Scale bar, 30 μm.

Supplementary Video 5 TPIM showing contact between microglial processes and neutrophils in inflamed brain parenchyma. Green, microglia (GFP); Red, neutrophils (Ab Ly6G); Blue, blood vessels (Lectin). Scale bar, 40 μm.

Supplementary Video 6 TPIM showing elongation of microglial processes toward point of microglia-neutrophil contact (white arrow). Green, microglia (GFP); Red, neutrophils (Ab Ly6G); Blue, blood vessels (Lectin).

Supplementary Video 7 TPIM showing neutrophil crawling along the perivascular region in inflamed brain parenchyma. Green, neutrophil (GFP); Red, blood vessels (Texas Red dextran); White, tracking of neutrophil. Scale bar, 50 μm.

Supplementary Video 8 TPIM showing neutrophil reverse-transendothelial migration from brain parenchyma into blood vessel. Green, neutrophil (GFP); Red, blood vessels (Texas Red dextran); White, tracking of neutrophil; Blue, the replacement of reverse-migrated neutrophil. Scale bar, 20 μm.

## Abbreviations

BBB: Blood-brain barrier
CNS: Central nervous system
GFP: Green fluorescent protein
LPS: Lipopolysaccharide
rTEM: reverse Transendothelial migration
SPF: Specific pathogen-free
TEM: Transendothelial migration
WGA: Wheat-germ agglutinin.

## References

[B1] Ahn S. Y. Maeng Y. S. Kim Y. R. Choe Y. H. Hwang H. S. Hyun Y. M. (2020). In vivo monitoring of dynamic interaction between neutrophil and human umbilical cord blood-derived mesenchymal stem cell in mouse liver during sepsis. *Stem. Cell Res. Ther.* 11:44. 10.1186/s13287-020-1559-4 PMC6998265 32014040

[B2] Baik S. H. Cha M. Y. Hyun Y. M. Cho H. Hamza B. Kim D. K. (2014). Migration of neutrophils targeting amyloid plaques in Alzheimer’s disease mouse model. *Neurobiol. Aging* 35 1286–1292. 10.1016/j.neurobiolaging.2014.01.003 24485508 PMC4248665

[B3] Brinkmann V. Zychlinsky A. (2012). Neutrophil extracellular traps: is immunity the second function of chromatin? *J. Cell Biol.* 198 773–783. 10.1083/jcb.201203170 22945932 PMC3432757

[B4] Buckley C. D. Ross E. A. McGettrick H. M. Osborne C. E. Haworth O. Schmutz C. (2006). Identification of a phenotypically and functionally distinct population of long-lived neutrophils in a model of reverse endothelial migration. *J. Leukoc. Biol.* 79 303–311. 10.1189/jlb.0905496 16330528

[B5] Burn T. Alvarez J. I. (2017). Reverse transendothelial cell migration in inflammation: to help or to hinder? *Cell Mol. Life Sci.* 74 1871–1881. 10.1007/s00018-016-2444-2 28025672 PMC11107488

[B6] Cai Z. Hussain M. D. Yan L. J. (2014). Microglia, neuroinflammation, and beta-amyloid protein in Alzheimer’s disease. *Int. J. Neurosci.* 124 307–321. 10.3109/00207454.2013.833510 23930978

[B7] Capucetti A. Albano F. Bonecchi R. (2020). Multiple roles for chemokines in neutrophil biology. *Front. Immunol.* 11:1259. 10.3389/fimmu.2020.01259 PMC7363767 32733442

[B8] Cheng O. Z. Palaniyar N. (2013). NET balancing: a problem in inflammatory lung diseases. *Front. Immunol.* 4:1. 10.3389/fimmu.2013.00001 PMC3553399 23355837

[B9] Christy A. L. Walker M. E. Hessner M. J. Brown M. A. (2013). Mast cell activation and neutrophil recruitment promotes early and robust inflammation in the meninges in EAE. *J. Autoimmun.* 42 50–61. 10.1016/j.jaut.2012.11.003 23267561

[B10] Colom B. Bodkin J. V. Beyrau M. Woodfin A. Ody C. Rourke C. (2015). Leukotriene B4-neutrophil elastase axis drives neutrophil reverse transendothelial cell migration In Vivo. *Immunity* 42 1075–1086. 10.1016/j.immuni.2015.05.010 26047922 PMC4504024

[B11] Davis B. M. Salinas-Navarro M. Cordeiro M. F. Moons L. De Groef L. (2017). Characterizing microglia activation: a spatial statistics approach to maximize information extraction. *Sci. Rep.* 7:1576. 10.1038/s41598-017-01747-8 PMC5431479 28484229

[B12] Ebersoldt M. Sharshar T. Annane D. (2007). Sepsis-associated delirium. *Intensive Care Med.* 33 941–950. 10.1007/s00134-007-0622-2 17410344

[B13] Echeverria V. Yarkov A. Aliev G. (2016). Positive modulators of the alpha7 nicotinic receptor against neuroinflammation and cognitive impairment in Alzheimer’s disease. *Prog. Neurobiol.* 144 142–157. 10.1016/j.pneurobio.2016.01.002 26797042

[B14] Elks P. M. van Eeden F. J. Dixon G. Wang X. Reyes-Aldasoro C. C. (2011). Activation of hypoxia-inducible factor-1alpha (Hif-1alpha) delays inflammation resolution by reducing neutrophil apoptosis and reverse migration in a zebrafish inflammation model. *Blood* 118 712–722. 10.1182/blood-2010-12-324186 21555741

[B15] Faust N. Varas F. Kelly L. M. Heck S. Graf T. (2000). Insertion of enhanced green fluorescent protein into the lysozyme gene creates mice with green fluorescent granulocytes and macrophages. *Blood* 96 719–726. 10.1182/blood.v96.2.719 10887140

[B16] Fortin M. Steff A. M. Hugo P. (2005). High-throughput technology: green fluorescent protein to monitor cell death. *Methods Mol. Med.* 110 121–137. 10.1385/1-59259-869-2:121 15901932

[B17] Girbl T. Lenn T. Perez L. Rolas L. Barkaway A. Thiriot A. (2018). Distinct compartmentalization of the chemokines CXCL1 and CXCL2 and the atypical receptor ACKR1 determine discrete stages of neutrophil diapedesis. *Immunity* 49 1062–1076.e6. 30446388 10.1016/j.immuni.2018.09.018 PMC6303217

[B18] Havenar-Daughton C. Lindqvist M. Heit A. Wu J. E. Reiss S. M. Kendric K. (2016). CXCL13 is a plasma biomarker of germinal center activity. *Proc. Natl. Acad. Sci. U.S.A.* 113 2702–2707. 10.1073/pnas.1520112113 26908875 PMC4790995

[B19] He H. Geng T. Chen P. Wang M. Hu J. Kang L. (2016). NK cells promote neutrophil recruitment in the brain during sepsis-induced neuroinflammation. *Sci. Rep.* 6:27711. 10.1038/srep27711 PMC4897692 27270556

[B20] Jiang W. St-Pierre S. Roy P. Morley B. J. Hao J. Simard A. R. (2016). Infiltration of CCR2+Ly6Chigh proinflammatory monocytes and neutrophils into the central nervous system is modulated by nicotinic acetylcholine receptors in a model of multiple sclerosis. *J. Immunol.* 196 2095–2108. 10.4049/jimmunol.1501613 26810225 PMC4760232

[B21] Jin H. Aziz M. Ode Y. Wang P. (2019). CIRP Induces neutrophil reverse transendothelial migration in sepsis. *Shock* 51 548–556. 10.1097/shk.0000000000001257 30148763 PMC6387861

[B22] Joly E. Hudrisier D. (2003). What is trogocytosis and what is its purpose? *Nat. Immunol.* 4:815. 10.1038/ni0903-815 12942076

[B23] Jung S. Aliberti J. Graemmel P. Sunshine M. J. Kreutzberg G. W. Sher A. (2000). Analysis of fractalkine receptor CX(3)CR1 function by targeted deletion and green fluorescent protein reporter gene insertion. *Mol. Cell Biol.* 20 4106–4114. 10.1128/mcb.20.11.4106-4114.2000 10805752 PMC85780

[B24] Kanashiro A. Hiroki C. H. da Fonseca D. M. Birbrair A. Ferreira R. G. Bassi G. S. (2020). The role of neutrophils in neuro-immune modulation. *Pharmacol. Res.* 151:104580. 10.1016/j.phrs.2019.104580 31786317 PMC7023896

[B25] Kettenmann H. Hanisch U. K. Noda M. Verkhratsky A. (2011). Physiology of microglia. *Physiol. Rev.* 91 461–553. 21527731 10.1152/physrev.00011.2010

[B26] Kobayashi Y. (2008). The role of chemokines in neutrophil biology. *Front. Biosci.* 13:2400–2407. 10.2741/2853 17981721

[B27] Kozlowski C. Weimer R. M. (2012). An automated method to quantify microglia morphology and application to monitor activation state longitudinally in vivo. *PLoS One* 7:e31814. 10.1371/journal.pone.0031814 22457705 PMC3294422

[B28] Kruger P. Saffarzadeh M. Weber A. N. Rieber N. Radsak M. von Bernuth H. (2015). Neutrophils: between host defence, immune modulation, and tissue injury. *PLoS Pathog.* 11:e1004651. 10.1371/journal.ppat.1004651 25764063 PMC4357453

[B29] Lee S. H. Choe Y. H. Kang R. H. Kim Y. R. Kim N. H. Kang S. (2019). A bright blue fluorescent dextran for two-photon in vivo imaging of blood vessels. *Bioorg. Chem.* 89:103019. 10.1016/j.bioorg.2019.103019 31176238

[B30] Li W. W. Setzu A. Zhao C. Franklin R. J. (2005). Minocycline-mediated inhibition of microglia activation impairs oligodendrocyte progenitor cell responses and remyelination in a non-immune model of demyelination. *J. Neuroimmunol.* 158 58–66. 10.1016/j.jneuroim.2004.08.011 15589038

[B31] Liu B. Hong J. S. (2003). Role of microglia in inflammation-mediated neurodegenerative diseases: mechanisms and strategies for therapeutic intervention. *J. Pharmacol. Exp. Ther.* 304 1–7. 10.1124/jpet.102.035048 12490568

[B32] Liu Y. W. Li S. Dai S. S. (2018). Neutrophils in traumatic brain injury (TBI): friend or foe? *J. Neuroinflam.* 15:146. 10.1186/s12974-018-1173-x PMC5960133 29776443

[B33] Ly N. P. Komatsuzaki K. Fraser I. P. Tseng A. A. Prodhan P. Moore K. J. (2005). Netrin-1 inhibits leukocyte migration in vitro and in vivo. *Proc. Natl. Acad. Sci. U.S.A.* 102 14729–14734. 10.1073/pnas.0506233102 16203981 PMC1253572

[B34] Mammana S. Fagone P. Cavalli E. Basile M. S. Petralia M. C. Nicoletti F. (2018). The role of macrophages in neuroinflammatory and neurodegenerative pathways of Alzheimer’s disease, amyotrophic lateral sclerosis, and multiple sclerosis: pathogenetic cellular effectors and potential therapeutic targets. *Int. J. Mol. Sci.* 19:831. 10.3390/ijms19030831 29533975 PMC5877692

[B35] Mathias J. R. Perrin B. J. Liu T. X. Kanki J. Look A. T. Huttenlocher A. (2006). Resolution of inflammation by retrograde chemotaxis of neutrophils in transgenic zebrafish. *J. Leukoc. Biol.* 80 1281–1288. 10.1189/jlb.0506346 16963624

[B36] Nathan C. (2006). Neutrophils and immunity: challenges and opportunities. *Nat. Rev. Immunol.* 6 173–182. 10.1038/nri1785 16498448

[B37] Neumann J. Henneberg S. von Kenne S. Nolte N. Muller A. J. Schraven B. (2018). Beware the intruder: real time observation of infiltrated neutrophils and neutrophil-Microglia interaction during stroke in vivo. *PLoS One* 13:e0193970. 10.1371/journal.pone.0193970 29543836 PMC5854356

[B38] Neumann J. Sauerzweig S. Ronicke R. Gunzer F. Dinkel K. Ullrich O. (2008). Microglia cells protect neurons by direct engulfment of invading neutrophil granulocytes: a new mechanism of CNS immune privilege. *J. Neurosci.* 28 5965–5975. 10.1523/jneurosci.0060-08.2008 18524901 PMC6670327

[B39] Park S. A. Choe Y. H. Park E. Hyun Y. M. (2018). Real-time dynamics of neutrophil clustering in response to phototoxicity-induced cell death and tissue damage in mouse ear dermis. *Cell Adh. Migr.* 12 424–431. 29733749 10.1080/19336918.2018.1471322 PMC6363031

[B40] Pillay J. Kamp V. M. van Hoffen E. Visser T. Tak T. Lammers J. W. (2012). A subset of neutrophils in human systemic inflammation inhibits T cell responses through Mac-1. *J. Clin. Invest.* 122 327–336. 10.1172/jci57990 22156198 PMC3248287

[B41] Podjaski C. Alvarez J. I. Bourbonniere L. Larouche S. Terouz S. Bin J. M. (2015). Netrin 1 regulates blood-brain barrier function and neuroinflammation. *Brain* 138 1598–1612. 10.1093/brain/awv092 25903786 PMC4614143

[B42] Ransohoff R. M. Kivisakk P. Kidd G. (2003). Three or more routes for leukocyte migration into the central nervous system. *Nat. Rev. Immunol.* 3 569–581. 10.1038/nri1130 12876559

[B43] Ransohoff R. M. Perry V. H. (2009). Microglial physiology: unique stimuli, specialized responses. *Annu. Rev. Immunol.* 27 119–145. 10.1146/annurev.immunol.021908.132528 19302036

[B44] Robertson A. L. Holmes G. R. Bojarczuk A. N. Burgon J. Loynes C. A. Chimen M. (2014). A zebrafish compound screen reveals modulation of neutrophil reverse migration as an anti-inflammatory mechanism. *Sci. Transl Med.* 6:225ra29. 10.1126/scitranslmed.3007672 24574340 PMC4247228

[B45] Rosenberger P. Schwab J. M. Mirakaj V. Masekowsky E. Mager A. Morote-Garcia J. C. (2009). Hypoxia-inducible factor-dependent induction of netrin-1 dampens inflammation caused by hypoxia. *Nat. Immunol.* 10 195–202. 10.1038/ni.1683 19122655

[B46] Saijo K. Glass C. K. (2011). Microglial cell origin and phenotypes in health and disease. *Nat. Rev. Immunol.* 11 775–787. 10.1038/nri3086 22025055

[B47] Sawant K. V. Poluri K. M. Dutta A. K. Sepuru K. M. Troshkina A. Garofalo R. P. (2016). Chemokine CXCL1 mediated neutrophil recruitment: Role of glycosaminoglycan interactions. *Sci. Rep.* 6:33123. 10.1038/srep33123 PMC5021969 27625115

[B48] Sevenich L. (2018). Brain-resident microglia and blood-borne macrophages orchestrate central nervous system inflammation in neurodegenerative disorders and brain cancer. *Front. Immunol.* 9:697. 10.3389/fimmu.2018.00697 PMC5897444 29681904

[B49] Shastri A. Bonifati D. M. Kishore U. (2013). Innate immunity and neuroinflammation. *Med. Inflamm.* 2013:342931. 10.1155/2013/342931 PMC3697414 23843682

[B50] Simmons S. B. Liggitt D. Goverman J. M. (2014). Cytokine-regulated neutrophil recruitment is required for brain but not spinal cord inflammation during experimental autoimmune encephalomyelitis. *J. Immunol.* 193 555–563. 10.4049/jimmunol.1400807 24913979 PMC4123857

[B51] Steff A. M. Fortin M. Arguin C. Hugo P. (2001). Detection of a decrease in green fluorescent protein fluorescence for the monitoring of cell death: an assay amenable to high-throughput screening technologies. *Cytometry* 45 237–243. 10.1002/1097-0320(20011201)45:4<237::aid-cyto10024>3.0.co;2-j 11746092

[B52] Tang C. Xue H. L. Bai C. L. Fu R. (2011). Regulation of adhesion molecules expression in TNF-alpha-stimulated brain microvascular endothelial cells by tanshinone IIA: involvement of NF-kappaB and ROS generation. *Phytother. Res.* 25 376–380. 20687137 10.1002/ptr.3278

[B53] Tauzin S. Starnes T. W. Becker F. B. Lam P. Y. Huttenlocher A. (2014). Redox and Src family kinase signaling control leukocyte wound attraction and neutrophil reverse migration. *J. Cell Biol.* 207 589–598. 10.1083/jcb.201408090 25488917 PMC4259815

[B54] Walcheck B. Kahn J. Fisher J. M. Wang B. B. Fisk R. S. Payan D. G. (1996). Neutrophil rolling altered by inhibition of L-selectin shedding in vitro. *Nature* 380 720–723. 10.1038/380720a0 8614468

[B55] Woodfin A. Voisin M. B. Beyrau M. Colom B. Caille D. Diapouli F. M. (2011). The junctional adhesion molecule JAM-C regulates polarized transendothelial migration of neutrophils in vivo. *Nat. Immunol.* 12 761–769. 10.1038/ni.2062 21706006 PMC3145149

[B56] Wu D. Zeng Y. Fan Y. Wu J. Mulatibieke T. Ni J. (2016). Reverse-migrated neutrophils regulated by JAM-C are involved in acute pancreatitis-associated lung injury. *Sci. Rep.* 6:20545. 10.1038/srep20545 PMC4740794 26841848

[B57] Xu Y. Feng D. Wang Y. Lin S. Xu L. (2008). Sodium tanshinone IIA sulfonate protects mice from ConA-induced hepatitis via inhibiting NF-kappaB and IFN-gamma/STAT1 pathways. *J. Clin. Immunol.* 28 512–519. 10.1007/s10875-008-9206-3 18498044

[B58] Yoo S. K. Huttenlocher A. (2011). Spatiotemporal photolabeling of neutrophil trafficking during inflammation in live zebrafish. *J. Leukoc. Biol.* 89 661–667. 10.1189/jlb.1010567 21248150 PMC3079246

[B59] Zenaro E. Pietronigro E. Della Bianca V. Piacentino G. Marongiu L. Budui S. (2015). Neutrophils promote Alzheimer’s disease-like pathology and cognitive decline via LFA-1 integrin. *Nat. Med.* 21 880–886. 10.1038/nm.3913 26214837

[B60] Zhao J. Bi W. Xiao S. Lan X. Cheng X. Zhang J. (2019). Neuroinflammation induced by lipopolysaccharide causes cognitive impairment in mice. *Sci. Rep.* 9:5790. 10.1038/s41598-019-42286-8 PMC6453933 30962497

[B61] Zhou H. Andonegui G. Wong C. H. Kubes P. (2009). Role of endothelial TLR4 for neutrophil recruitment into central nervous system microvessels in systemic inflammation. *J. Immunol.* 183 5244–5250. 10.4049/jimmunol.0901309 19786543

