# Supplementary figures and images for: Neutrophils Return to Bloodstream Through the Brain Blood Vessel After Crosstalk With Microglia During LPS-Induced Neuroinflammation
Source: Front Cell Dev Biol. 2020 Dec 8;8:613733. doi: 10.3389/fcell.2020.613733 (PMC7753044; doi:10.3389/fcell.2020.613733)

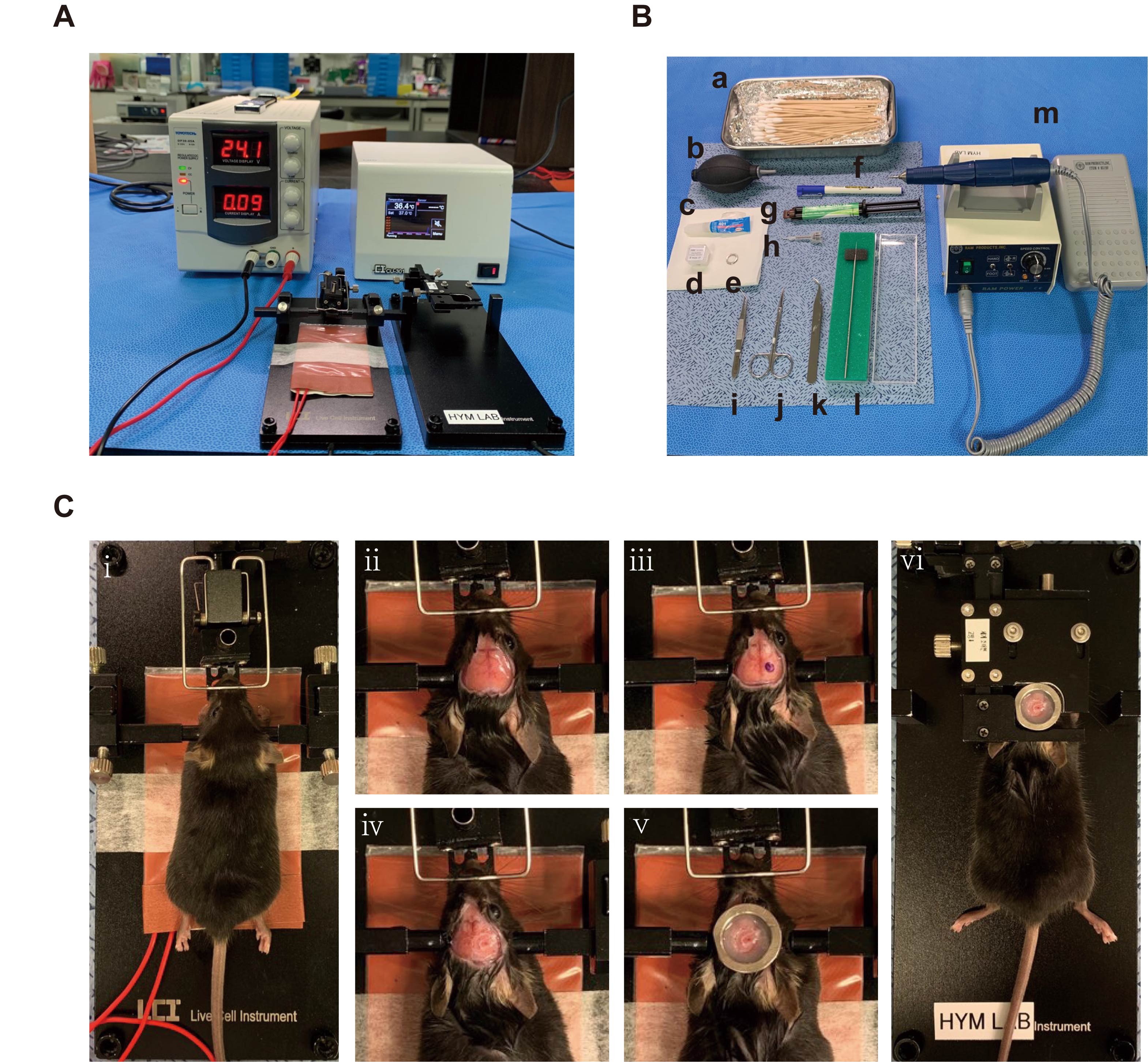

Supplement: Supplementary Figure 1 — Cranial window surgery setup for brain intravital imaging. (A) Brain chambers with heating plates for maintaining body temperature at 37 ± 0.5°C. a. Metal and rubber heating plates, b. DC temperature controller, c. Stereotactic instrument for cranial window surgery, d. Head fixation device for imaging. (B) Subsidiary tools for surgery a. cotton swab, b. air blower, c. tissue glue, d. cover glass (diameter = 5 μm), e. metal frame, f. pen, g. dental cement, h. resin tip, i. forceps, j. a pair of scissors, k. forceps, l. micro forceps, m. micro drill. (C) Surgical procedure of cranial window surgery for intravital brain imaging. i. Fix the mouse in a stereotactic heating plate, ii. Remove head skin and the periosteum, iii. Coordinate predestinate location carved on right hemisphere, iv. Moisturize a circular opening with a drop of isotonic saline solution and seal a circular opening with a 5 μm coverslip, v. Glue and fix the metal frame on the borders of the cranial window and skull area using dental cement, vi. Place the mouse on a head fixation device for intravital imaging. [file Image_1.JPEG]
